# Cardiac Involvement in Dystrophin-Deficient Females: Current Understanding and Implications for the Treatment of Dystrophinopathies

**DOI:** 10.3390/genes11070765

**Published:** 2020-07-08

**Authors:** Kenji Rowel Q. Lim, Narin Sheri, Quynh Nguyen, Toshifumi Yokota

**Affiliations:** 1 Department of Medical Genetics, Faculty of Medicine and Dentistry, University of Alberta, Edmonton, AB T6G2H7, Canada; kenjirow@ualberta.ca (K.R.Q.L.); narin@ualberta.ca (N.S.); nguyenth@ualberta.ca (Q.N.); 2 The Friends of Garrett Cumming Research & Muscular Dystrophy Canada HM Toupin Neurological Science Research Chair, Edmonton, AB T6G2H7, Canada

**Keywords:** Duchenne muscular dystrophy, Becker muscular dystrophy, female dystrophin mutation carriers, cardiac involvement, partial dystrophin deficiency, female carrier models, gene therapy

## Abstract

Duchenne muscular dystrophy (DMD) is a fatal X-linked recessive condition caused primarily by out-of-frame mutations in the dystrophin gene. In males, DMD presents with progressive body-wide muscle deterioration, culminating in death as a result of cardiac or respiratory failure. A milder form of DMD exists, called Becker muscular dystrophy (BMD), which is typically caused by in-frame dystrophin gene mutations. It should be emphasized that DMD and BMD are not exclusive to males, as some female dystrophin mutation carriers do present with similar symptoms, generally at reduced levels of severity. Cardiac involvement in particular is a pressing concern among manifesting females, as it may develop into serious heart failure or could predispose them to certain risks during pregnancy or daily life activities. It is known that about 8% of carriers present with dilated cardiomyopathy, though it may vary from 0% to 16.7%, depending on if the carrier is classified as having DMD or BMD. Understanding the genetic and molecular mechanisms underlying cardiac manifestations in dystrophin-deficient females is therefore of critical importance. In this article, we review available information from the literature on this subject, as well as discuss the implications of female carrier studies on the development of therapies aiming to increase dystrophin levels in the heart.

## 1. Introduction

Duchenne muscular dystrophy (DMD) is a fatal, X-linked recessive condition affecting 1:3500–5000 males worldwide [1,2]. It is characterized by progressive body-wide muscle weakness and wasting, causing patients to be wheelchair-bound by their teenage years. The disease progresses rapidly, and death occurs typically by the mid-twenties due to respiratory or cardiac complications [3,4,5]. The condition is due to mutations in the *DMD* gene, which codes for the dystrophin protein [1,3,4,6]. The majority of patients (≈60%) have large, out-of-frame deletions, where the resulting *DMD* mRNA is degraded, and dystrophin is not produced [3]. Dystrophin connects the cytoskeleton of muscle fibers via its N-terminal actin-binding domain with the surrounding extracellular matrix via its cysteine-rich C-terminal domain, acting as a stabilizing anchor [7]. Without dystrophin, muscle membrane integrity is lost and muscle degradation occurs [8]. Becker muscular dystrophy (BMD) is a milder disorder, characterized instead mostly by in-frame mutations in the *DMD* gene [9]. BMD is less common, affecting 1:18,500 new-born males [1]. Similar to DMD, the condition causes muscle weakness and wasting, though not to the same degree of severity. BMD patients exhibit slower disease progression as well as the ability to walk independently [10].

Both DMD and BMD are X-linked conditions, and so they usually affect males. About one-third of all DMD cases are caused by de novo mutations, with the other two-thirds due to inheritance from the mother [11]. This means that presumably, every mother of an isolated male DMD case has a two-thirds chance of being a carrier [12]. Even with a normal *DMD* gene in one chromosome, female carriers can present similar symptoms as affected males, showing muscle weakness, abnormal gait, fatigue, and cardiac involvement [13,14,15,16].

Cardiac symptoms are particularly prevalent in female dystrophin mutation carriers, affecting about 8% of this population with dilated cardiomyopathy (DCM) as a common presentation [13,15,17]. One review consolidated information from large studies and found that DCM was present in 7.3–16.7% of female DMD carriers and 0–13.3% of female BMD carriers [18]. The risk and severity of cardiomyopathy in female carriers increase with age and can have adverse effects as the condition worsens. In extreme cases, some may even need a heart transplant due to DCM-associated cardiac failure [10]. Corticosteroid use has generally been associated with better cardiac function and improved survival in male DMD patients, with the latter attributed to a decrease in heart failure-caused mortality [19,20,21,22]. However, the benefit of corticosteroids for female carrier cardiac involvement is currently unclear due to a lack of clinical studies on this population [18]. The same can be said for other cardiac interventions. Due to these concerns, it is imperative that we understand the unique circumstances of disease pathogenesis in female dystrophin-deficient patients. A survey of the current state of the field shows improved screening and diagnosis of female carriers [23], allowing for the timely and effective management of clinical conditions that may arise. There is also a bigger push now to understand the molecular mechanisms underlying disease progression and manifestation in females with dystrophin mutations. For instance, representative in vitro and in vivo models have been created and are available for these kinds of investigations. In this review, we will touch on the mentioned items and discuss the implications of what we can learn from female carriers for the treatment of DMD.

## 2. Clinical Presentation of Female Dystrophin Mutation Carriers

The onset of clinical manifestations for symptomatic female carriers is variable, ranging from early childhood to late adulthood [24]. In a study of 15 manifesting DMD carriers, the age of onset was found to be anywhere from age 2 to 47 [14]. The majority of cases become symptomatic during puberty [25]. Disease severity is variable and genotype–phenotype correlations are not well established in this group of patients [22,24]. Cardiac manifestation in DMD and BMD female carriers is mostly in the form of DCM. Other manifestations include conduction defects and arrhythmias, but these could be consequences of long-term DCM [18,22,24]. Acute heart failure and non-sustained ventricular tachycardia have been reported as initial presentations in late adulthood, although these are not as common [26,27]. Cardiac involvement may be present without concomitant skeletal muscle manifestations [24]. It is worth noting that while the cardiac manifestations in female carriers may be subclinical under normal physiological conditions, they can worsen and become symptomatic during major events such as pregnancy or birth [24]. DMD carriers are also prone to develop cardiac complications when under general anesthesia [24]. In one reported case of a female DMD carrier, the DCM was so severe as to require a heart transplant [28].

Imaging studies such as cardiac magnetic resonance imaging (cMRI) and echocardiography further characterized the structural and functional features of cardiac involvement in these patients. In accordance with DCM, left ventricular systolic dysfunction was present in up to 40% of DMD carriers and 6% of BMD carriers [18]. DCM appeared mostly in the left ventricular posterior wall. Myocardial fibrosis, indicated by late gadolinium enhancement (LGE) on cMRI, was detected in 35–65% of DMD carriers and in 19–20% of BMD carriers [18]. LGE patterns were similar between female carriers and their male relatives. Furthermore, left ventricular hypertrabeculation (LVHT) was reported in female carriers of dystrophinopathies [24]. A previous study found LVHT present in up to 40% of DMD carriers using cMRI [24]. LVHT carries with it serious implications due to the risk of heart failure, thromboembolism, ventricular arrhythmias, and sudden cardiac death.

Owing to the invasive and technically challenging nature of obtaining a cardiac biopsy, information on the histological features of these manifesting carriers are scarce. An endomyocardial biopsy from a clinically diagnosed DMD female carrier showed a mosaic expression pattern of dystrophin among cardiomyocytes (CMs) [29]. This finding is consistent with patterns seen in heterozygous canine and *mdx* mouse carriers, presumably due to X chromosome inactivation (XCI) in females [29,30,31,32].

Besides cardiac manifestations, female dystrophin mutation carriers can present with other systemic features. Limb girdle weakness, gait disturbance, exercise intolerance, calf hypertrophy, and scoliosis have all been recognized as skeletal muscle manifestations in these patients. Elevated serum creatinine kinase (CK) is often found in patients with skeletal muscle presentations. Additionally, neurocognitive problems can present as learning disabilities or behavioral problems in this patient population [33]. Despite being rare, a case of a homozygous BMD female was described previously in the literature, presenting with exercise intolerance and recurrent myoglobinuria as dominant features [34].

X-linked dilated cardiomyopathy (XLDC) is a unique subset of dystrophinopathy with its own distinct presentation. XLDC patients develop rapidly progressive cardiomyopathy without overt skeletal muscle disease [35,36,37]. Death often occurs between 10 and 20 years of age due to the acuity and severity of cardiac manifestations. In some instances, female carriers of XLDC may develop cardiomyopathy later on in life with reduced severity [35]. Elevated serum CK is often detected in both patients and carriers. Various disease-causing mutations in the *DMD* gene have been reported for XLDC, however, the precise relationship between these genetic changes and their distinct clinical presentations remains unclear [35]. The proposed mechanisms largely focus on differences in gene regulation, dystrophin stability, and protein–protein interactions between skeletal and cardiac muscles. Exercise overload-mediated cardiac damage due to being unaware of possible cardiac involvement in the absence of overt skeletal muscle disease in these patients has been proposed as another potential mechanism [36].

## 3. Mechanisms Underlying DMD/BMD Manifestation in Females

There are many ways a female carrier of a dystrophin mutation (or mutations) may manifest symptoms. We will divide them into three major categories: chromosomal aberrations, simple inheritance, and hormonal events. Manifesting carriers are often reported to be the result of XCI, but other mechanisms have been noted in individual cases. Most mechanisms result in a classic carrier status, meaning they are heterozygous for a *DMD* mutation. However, we will also discuss cases of manifesting females who have *DMD* mutations on both X chromosomes.

### 3.1. Chromosomal Aberrations

**Figure 1:**
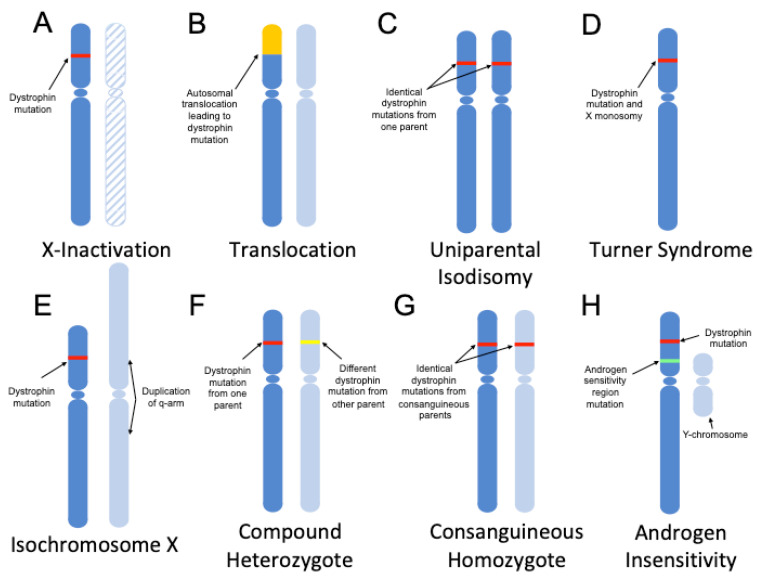
Genetic mechanisms of dystrophinopathy manifestation in females. Chromosomes from different parents are represented by different shades of blue, with the *DMD* mutation represented by a red or yellow line. Females with dystrophin mutations may manifest symptoms as a result of: (**A**) skewed X chromosome inactivation (the inactive chromosome is hashed); (**B**) balanced translocations with an autosome, represented in yellow, involving regions within the *DMD* gene; (**C**) uniparental isodisomy; (**D**) inheritance of a single *DMD* mutation-containing X-chromosome (a subcategory of Turner syndrome); (**E**) Xq isochromosome formation in tandem with a *DMD* mutation on the other X chromosome; (**F**) compound heterozygosity, with the inheritance of two different *DMD* mutations on either chromosome; (**G**) homozygous *DMD* mutation inheritance due to consanguinity; and (**H**) androgen insensitivity in an X/Y female (due to an androgen receptor mutation, represented by a green line) in tandem with a *DMD* mutation.

Chromosomal aberrations include any mutations or deviations to the normal chromosome structure in humans, such as having missing, extra, or irregular chromosomal portions. One of the most studied chromosomal events concerning female carriers is skewed XCI (Figure 1A). XCI typically happens randomly during early female development, with the expectation of an even 50:50 split in gene expression between the two X chromosomes [38]. In cases of skewed XCI however, there is usually a mutation in the *DMD* gene that causes an X chromosome with that variant to be preferentially activated over the other [39,40,41]. This means that the X chromosome containing the normal *DMD* gene is inactivated at a higher rate than the X chromosome that carries the mutation. This form of inheritance shows a mosaic pattern of dystrophin expression, causing some regions to be dystrophin-negative while others being dystrophin-positive. Skewed XCI is more common in carriers of X-linked mutations, however, the underlying mechanisms for the skewing are unclear [42]. Balanced translocations between the X chromosome and other chromosomes may also result in female carrier status (Figure 1B) [43,44]. In this form of inheritance, the translocation occurs within the *DMD* gene, causing a deletion mutation. It can theoretically happen with any autosomal chromosome and has been noted to occur with chromosomes 12 and 21 [43,44]. When XCI occurs, these balanced translocations can lead to similar mosaic patterns of dystrophin expression that are seen in female carriers of *DMD* mutations.

Other chromosomal aberrations include the event where both X chromosomes are inherited from one parent, called uniparental isodisomy. In these cases, a non-disjunction event occurs, typically during meiosis II, that causes both copies of a chromosome to be present in the gamete (Figure 1C) [45]. A case has been reported of an affected female who was homozygous for the same *DMD* gene mutation, pointing to uniparental isodisomy [46]. The 6-year-old female presented with classic DMD symptoms including muscle weakness of her upper and lower extremities. It was found that the homozygosity was not due to a meiosis II non-disjunction event, but rather from either a second non-disjunction event in the zygote or a duplication of the maternal chromosome in combination with a sperm that did not have either the X or Y chromosome. The inherited maternal X chromosome carried the mutant *DMD* gene, causing the proband to be homozygous for the same deletion in exon 50 on both X chromosomes. Immunochemistry results from a muscle biopsy showed virtually no dystrophin production, indicating a lack of mosaicism [46].

Sex chromosome monosomy is another way a female carrier may manifest dystrophinopathy symptoms (Figure 1D). There has been a report of a female with Turner syndrome (X monosomy) who carried a de novo *DMD* mutation [47]. In cases of Turner Syndrome, there is only one X chromosome, so there is no XCI that occurs. With only one X chromosome, any X-linked mutations present will affect the individual as though they were homozygous for the mutation. In this case, both clinical signs of Turner syndrome and DMD were present, with the proband being wheelchair-bound by age 9 [47]. Cases of Turner syndrome are typically monosomic, but about 5–10% have been linked to isochromosome Xq (i(Xq)) [48]. Isochromosomes occur when one arm of a chromosome is duplicated, while the other is deleted (Figure 1E). A case has been reported of a female with i(Xq) and a de novo *DMD* mutation [49]. The proband showed clinical manifestations of DMD and Turner syndrome early in life. Due to a deletion of the X-chromosome p-arm, individuals with i(Xq) are hemizygous for all genes on the p-arm, which includes the *DMD* gene [49].

### 3.2. Simple Inheritance

Females typically carry two X chromosomes, which means it is possible to have two different mutations in each chromosome separately. These cases are referred to as compound heterozygotes—two different mutations acting together to produce a phenotype akin to homozygous-mutant individuals (Figure 1F). In these cases, the disorder is phenotypically identical to classic DMD/BMD because no matter which X chromosome is inactivated during XCI, there will always either be a mutant protein produced or no protein produced at all. A case has been noted of a compound heterozygote female with severe BMD symptoms and dilated cardiomyopathy at age 15 [14]. The two mutations, in this case, were a deletion of exons 8–13 and an intron 69 splice site mutation (c.10086 + 2T > C). Both mutations were likely due to de novo mutations occurring in the germline, as her mother was not a carrier of either mutation.

There has also been a reported case of a female with BMD who had both X chromosomes carrying the same dystrophin mutation through consanguinity (Figure 1G) [34]. In this case, the individual’s parents were linked through a common great-grandfather. The patient was found to be homozygous for an in-frame deletion in exons 44–45, with identical deletions found in both parents. Interestingly, this family had reported multiple cases of cardiac disease, with a few males having died from cardiomyopathy. In both of these scenarios, we see BMD phenotypes from the inheritance of two mutated copies of the mutated genes: one being a compound heterozygote with two different mutations, and the other being homozygous for the same mutation due to consanguinity. There has been one reported case of a female with severe DMD born to consanguineous parents [16]. The proband passed away from heart failure when she was 13.

### 3.3. Hormonal Events

Complete androgen insensitivity in combination with a *DMD* gene mutation may also lead to a dystrophic phenotype in X/Y females (Figure 1H) [50]. In a case seen by Katayama et al., an X/Y female was reported that had a de novo dystrophin mutation along with a mutation in the androgen receptor gene on Xq11-q12. The latter mutation caused androgen insensitivity, which can repress male genital formation as well as the development of secondary sex characteristics [51]. This led to a female phenotype in the individual or, more specifically, male pseudohermaphroditism [52]. The proband’s sister also presented a female X/Y phenotype having the same androgen sensitivity mutation, with the mother being a carrier of the mutation. The *DMD* mutation, a deletion of exons 12–19, was de novo as neither parent was a carrier. The co-occurrence of these two mutations led to a female DMD phenotype. As the proband only has one X chromosome, classical DMD symptoms were shown including muscle weakness and the inability to stand up independently.

## 4. Cardiac Phenotypes of Cellular and Female Animal Dystrophinopathy Models

### 4.1. Cellular Models

Human induced pluripotent stem cells (hiPSCs) have been extensively used to model DMD and BMD patient mutations [53,54]. In tandem with genome editing technologies such as transcription activator-like effector nucleases (TALENs) or the clustered regularly interspaced short palindromic repeats (CRISPR) system, virtually any dystrophin mutation can be recreated in hiPSCs. Differentiating these cells into CMs offers the unique opportunity to study cardiac pathology in vitro, and to evaluate the efficacy of DMD therapies in this context [55,56].

hiPSCs from female dystrophin mutation carriers have been developed. Tchieu et al. (2010) reprogrammed fibroblasts from an asymptomatic female carrier of a *DMD* exons 46–50 deletion using a polycistronic lentiviral vector with *OCT4*, *KLF4*, *SOX2*, and *c-MYC* [57]. Clonal lines were produced, which either expressed only the wild-type or the mutant *DMD* gene. This indicates that the resulting hiPSCs retained the XCI status of their respective parent somatic cell. A succeeding study by Miyagoe-Suzuki et al. (2017), however, shows that hiPSC production for female carriers is not as simple as initially thought. In their report, fibroblasts from a manifesting female carrier of a *DMD* exons 42–43 deletion were reprogrammed via two strategies: (1) using a polycistronic retroviral vector with *OCT4*, *KLF4*, *SOX2*, *LIN28*, and *NANOG*, or (2) using a cocktail of Sendai viral vectors that individually carried *OCT4*, *KLF4*, *SOX2*, and *c-MYC* [58]. Founder fibroblasts had skewed XCI, favoring inactivation of the chromosome with the wild-type *DMD* allele. Both strategies produced hiPSCs that retained this skewed XCI status. Interestingly however, the majority of hiPSC lines generated (three of five from strategy 1 and three of four from strategy 2) showed equal expression from both X chromosomes, indicating X chromosome reactivation (XCR). Differentiation of XCR lines into skeletal muscle cells showed dystrophin expression similar to wild-type controls by immunostaining. Molecular analysis revealed equal expression of both wild-type and mutant alleles in these cells, indicating the occurrence of random XCI following differentiation.

One study evaluated the utility of hiPSCs for modelling female DMD CMs. Eisen et al. (2019) generated hiPSCs from the fibroblasts of a manifesting female carrier with a *DMD* exons 8–12 deletion [59]. Reprogramming was done with Sendai viral vectors as described above. The authors similarly observed apparent XCR based on the loss of *XIST* RNA expression in hiPSCs. Upon differentiation into CMs, mixed expression of wild-type and mutant alleles was observed, which suggested the initiation of random XCI. Female DMD hiPSC-CMs exhibited an array of electrophysiological abnormalities, including evidence of arrhythmia. It would be helpful to further characterize the phenotype of female carrier hiPSC-derived CMs in other aspects, e.g., structure or contractile performance, while taking XCI status into account.

At this point, the factors influencing XCI stability upon cellular reprogramming remain unclear. Reports describing attempts to generate female carrier hiPSC models for other diseases reveal a similar situation [59,60,61]. There is also the case of XCI erosion to consider, in which an inactive X chromosome becomes activated as hiPSCs are cultured for long periods of time [62]. A more relevant concern would be to know how these events determine which X chromosome stays active upon differentiation of hiPSCs to CMs. If XCR were active, it appears this is usually resolved by random XCI in CMs [58,59,61]—In which case, we are limited to modelling CM populations that are approximately 50% wild-type and 50% mutant. This does not faithfully represent female carriers with skewed XCI, for instance. Ideally, generated hiPSC-derived CM populations should reflect the mosaic dystrophin expression levels observed in the heart of female carriers. However, knowing these native mosaicism levels in patients is a challenge in itself, since cardiac biopsies are not practical to obtain. More research into how we can perform such estimates is needed. At the moment at least, determining dystrophin mosaicism levels is recommended when using CMs induced from female carrier hiPSCs to aid in the careful interpretation of results. The creation of standardized reprogramming and differentiation protocols would help maintain consistency within and between studies.

Other cell-based models can be explored. An example would be the use of hiPSC-based CMs to generate 3D engineered heart muscle (EHM), allowing for a better representation of human myocardial tissue in vitro. Long et al. (2018) created mosaic EHM models by mixing CMs derived from DMD and CRISPR-corrected DMD hiPSCs at various ratios [63]. This revealed that the presence of at least 30% repaired CMs was sufficient to partially improve the contractile properties of dystrophic EHMs. As this method allows for more precise control of mosaicism levels in vitro, perhaps it could be adapted for studying cardiac phenotypes associated with varying amounts of dystrophic and healthy CMs, as opposed to those of non-treated and treated dystrophic CMs.

### 4.2. Animal Models

A number of animal models for female dystrophin mutation carriers have been studied, yielding insights into the cardiac phenotype seen in these patients. All carrier mouse models are based on the widely used *mdx* background (Table 1). *Mdx* mice harbor a spontaneous nonsense mutation in *Dmd* exon 23, resulting in a loss of full-length dystrophin protein synthesis [64]. The most basic model would be the female *mdx*/X heterozygote. Studies confirm that the hearts of these mice have ≈51% to 58% dystrophin-positive CMs based on immunostaining [30,65,66], slightly above what is expected from random XCI. On the C57BL/10ScSnJ (BL10) background, female *mdx*/X heterozygote cardiac structure and function are mostly indistinguishable from wild-type mice regardless of age, as evaluated by gross anatomical measurements, histology, electrocardiography (ECG), and pressure-volume (PV) loop analysis [30,65]. Expression levels of myomiRs, a set of microRNAs elevated in DMD, were not affected in these mice, at least at 2 months old [67]. Stress induction with β-isoproterenol or dobutamine mostly revealed no differences with wild-type controls [30,65]. Interestingly in one study, single or repeated injections of a high β-isoproterenol dose (10 mg/kg; the previous study used 0.35 mg/kg [30]) led to 21% or 31% mortality, respectively, in 4–6-month-old carrier mice that inherited the *mdx* mutation from the mother [66]. Carriers that inherited the *mdx* mutation paternally all survived, implying that additional epigenetic or environmental factors are affecting the response of these mice to cardiac stress.

The results above indicate that mice with ≈50% dystrophin-positive CMs in the heart do not show dystrophic cardiac phenotypes under normal or stressful conditions. It would be informative though to study phenotypes associated with varying compositions of dystrophin-positive CMs in the heart, as is likely exhibited by the female carrier population. This is achieved by the female *mdx*-*Xist*^Δhs^ mouse model, which has the *mdx* mutation on one X chromosome, and a *Xist* promoter mutation on the other [68]. The *Xist* promoter mutation skews XCI so that the chromosome bearing this mutant allele (which also has the wild-type *Dmd* gene) is preferentially inactivated at varying frequencies [68,76]. Thus, a spectrum of *mdx*-*Xist*^Δhs^ mice can be produced that each has different amounts of dystrophin-expressing CMs in the heart.

Studies on *mdx*-*Xist*^Δhs^ mice (mixed BL10-C57BL/6J [B6]/background) with 3–15% of healthy dystrophin levels by Western blotting revealed that left ventricle ejection fraction (EF) and stroke volume (SV) were similar in these mice on average to wild-type controls at 2, 6, and 10 months of age [69]. At 6 and 10 months, these values were significantly higher compared to homozygous female *mdx* mice. When 10-month-old *mdx*-*Xist*^Δhs^ mice were grouped into those having <4% or >4% dystrophin levels in the heart, those belonging to the <4% group had similar EF in both ventricles as age-matched *mdx* mice. Mice in the >4% group on the other hand had similar EF to wild-type, supporting a correlation between dystrophin levels and systolic function. Cardiac fibrosis was not detected at 2 months, but was significantly increased compared to healthy controls at 6 and 10 months in *mdx*-*Xist*^Δhs^ mice; at 10 months, however, it was lower by around 45% compared to female *mdx* mice. Heart wall and interventricular septum thicknesses, as well as heart rate, were not affected in the 3–15% *mdx*-*Xist*^Δhs^ mice versus wild-type. These results show that having as low as 4% mosaic dystrophin expression in the heart could ameliorate dystrophic cardiac pathology in mice.

Utrophin is known to compensate for dystrophin in *mdx* mice, leading to less severe phenotypes than those seen in patients [77,78]. The effect of knocking out utrophin in *mdx*-*Xist*^Δhs^ mice was therefore studied. *Mdx*/*utrn*^−/−^/*Xist*^Δhs^ mice (highly mixed background) at 10 months were divided into low (2.5–10.9%) and high (16.4–49.2%) dystrophin-expressing groups, with levels determined by Western blotting of heart samples [70]. Unlike *mdx*-*Xist*^Δhs^ mice, left ventricle EF was reduced to 32.4% and 50.7% for the low and high groups, respectively, compared to wild-type controls at 58.4%. SV was significantly decreased in both groups of *mdx*/*utrn*^−/−^/*Xist*^Δhs^ mice. Cardiac fibrosis was significantly increased in these mice, similar to what was seen in *mdx*-*Xist*^Δhs^ mice but at higher levels regardless of belonging to the low or high dystrophin group. In addition, also important to consider is that *mdx*/*utrn*^−/−^/*Xist*^Δhs^ mice exhibited decreased survival depending on their dystrophin expression level, with only 62% of those having <4% remaining alive at 6 months. Clearly, the knockout of utrophin worsened the phenotype of *mdx*-*Xist*^Δhs^ mice, even at dystrophin levels above the previously studied 3–15%. Whether this is more representative of the female carrier phenotype remains to be determined, given that upregulated utrophin expression is naturally observed in the dystrophin-negative fibers of carrier skeletal muscle biopsies [79].

Mosaic dystrophin expression in vivo can also be achieved through the injection of *mdx* embryonic stem cells (ESCs) into wild-type blastocysts. Interestingly, chimeric progeny with 30% or 50% *mdx* CMs showed significantly elevated cardiac fibrosis at 10 months [71]. Reduced left ventricle EF was observed by echocardiography in these mice at 16–18 months, at levels similar to *mdx* mice and ≈15% less than wild-type. Chimeras with less than 5% *mdx* incorporation were mostly similar to wild-type for these parameters. These findings are in stark contrast to what was observed in female *mdx*/X heterozygotes or *mdx*-*Xist*^Δhs^ mice. Particularly for the progeny with 50% *mdx* CMs, it is interesting why they did not show a wild-type phenotype similar to *mdx*/X mice, which had normal cardiac pathology even at 21 months of age [65]. It is suggested that ESC injection may be inducing so-called neomorphic effects, resulting in unique phenotypes in the course of development [71]. Given these findings, it would be informative to know if the reverse, i.e., injecting wild-type ESCs into *mdx* blastocysts [80], will produce different results with respect to cardiac phenotype.

All mouse models discussed so far have had mosaic dystrophin expression, but there are also those with uniformly low or no levels of dystrophin in the heart. While such models do not represent carriers per se, these would help understand cardiac pathology in females with dystrophin gene mutations on both chromosomes [14,34]. Due to XCI, one would expect that the cardiac involvement in non-mosaic female models would be similar to those found in male dystrophic mice. However, one study reported that at 22 months female *mdx* homozygotes had more severe left ventricle hemodynamic function than male *mdx* mice, as evaluated by PV loop analysis [72]. At this age, left ventricle EF was also significantly reduced in female *mdx* mice compared to both wild-type controls and male *mdx* mice. ECG, hydroxyproline content (a marker for fibrosis), and lifespan showed no sex differences, but female *mdx* mice appeared to have heavier hearts than male *mdx* mice when normalized to body weight [72,73]. These aforementioned experiments were done on *mdx* mice on the BL10 background. On the DBA/2J.B10 (D2) background, the opposite was observed for heart weight: 8-month-old male *mdx* mice had significantly heavier hearts than age-matched female *mdx* mice [74]. These findings suggest that sex and genetic background both modify the cardiac phenotype in *mdx* mice, likely through complex interactions at the molecular level.

It would be intriguing but challenging to compare this with the situation in humans, given that huge differences in clinical severity exist between male and female dystrophinopathy patients. One study evaluated female BMD/DMD carriers and their first-degree male BMD/DMD relatives via cMRI [81]. Pathological cMRI findings were significantly more common in males than female carriers by a factor of three. Myocardial fibrosis patterns were similar between males and female carriers that presented with cardiac pathology. There are of course a host of other factors to consider such as age (males were significantly younger than the female carriers in the study), anatomy, physiology, genetic background, and environment that make comparisons difficult between the mouse and human studies. Conducting more clinical comparative studies may help broaden our understanding of these sex-specific differences in humans. Research into modifier genes responsible for such differences would likewise be informative [82].

On the other hand, studies on female *mdx*^3cv^ mice (B6 background), which carry a splicing-disruptive point mutation in *Dmd* intron 65 [83] and have ≈3.3% dystrophin of wild-type levels in the heart, revealed that uniform low-level dystrophin expression does not improve gross anatomical and histopathological features of the heart compared to female dystrophin-null *mdx*^4cv^ mice [75]. *Mdx*^4cv^ mice have a nonsense point mutation in *Dmd* exon 53, introducing a premature stop codon that prevents dystrophin synthesis [84]. ECG showed improvements in female *mdx*^3cv^ mice but were mostly non-significant. Some measures of diastolic function were also restored to wild-type levels in these mice; EF, SV, and cardiac output remained significantly reduced and similar to those seen in *mdx*^4cv^ mice. Comparisons between female and male *mdx*^3cv^ mice were not performed but would have been useful to see sex-specific differences in the context of uniformly low dystrophin expression in the heart. A study on female utrophin-null *mdx*^3cv^ mice is also available, however cardiac phenotypes were not assessed [85].

Female dystrophinopathy carriers in other animals have been explored as well but at a limited depth. Gaschen et al. (1999) studied the cardiac phenotype of dystrophin-deficient cats [86]. At 9 months of age, female carriers had similar echocardiographic parameters as wild-type, except for the left ventricular internal diameter (LVID) which was significantly reduced during both systole and diastole; this difference normalized at 12 months. No clinical signs of heart failure were observed for female carriers or affected females, from 3 to 12 months of age. Moise et al. (1991) examined golden retrievers with muscular dystrophy [87]. A subset of female carrier dogs showed ECG lesions, whereas the rest were indistinguishable from wild-type; heart rate was higher in female carriers compared to wild-type on average, however. One carrier presented with a mild systolic murmur. Of the 11 female carriers studied, six had hyperechoic lesions from echocardiography that indicated calcification in the myocardium or fibrosis. These lesions were less severe compared to affected dogs. Affected dogs were evaluated as a combination of males and females in this study, and so observations exclusively on affected females could not be done. A more recent study by Kane et al. (2013) with golden retriever muscular dystrophy carriers (6–38 months of age) showed no differences with wild-type females in terms of systolic function as per echocardiography, but did confirm the presence of ECG abnormalities in the majority of carriers [32]. Increased study of female carriers in other animal models is definitely recommended, to allow for comparisons with phenotypes seen in mouse and human female carriers.

## 5. Implications for Therapy: How Much Dystrophin Is Enough for the Heart?

A longstanding question in the field is to know what amount of dystrophin in the heart is sufficient to prevent or at least ameliorate the cardiac symptoms observed in DMD. From what we know about human female carriers and studies on female *mdx*/X heterozygote mice, it appears that having 50% dystrophin-positive CMs in the heart typically leads to an asymptomatic or very mild cardiac presentation. This is confirmed at the cellular level by experiments using hiPSC-derived EHMs [63]. As we have also learned from female carrier mouse models, even as low as 4% mosaic expression of dystrophin in the heart could improve or at the very least provide some protection against dystrophin-deficient cardiac pathology [69].

This latest observation shows that low to moderate levels of dystrophin in the heart can have beneficial effects on cardiac function. This is encouraging since certain therapies being developed for DMD such as exon skipping with antisense oligonucleotides (AOs) typically have limited efficacy in the heart [88]. Some points from the animal studies have to be recognized, however. Firstly, the carrier mice studied either had mosaic or uniformly low-level dystrophin expression in the heart since early development (Table 1). While findings may be applicable in the case of female dystrophin mutation carriers, it may not be the case for patients receiving dystrophin-increasing therapies some years after birth. For example, one study evaluated serum myomiR levels in *mdx*-*Xist*^Δhs^ and exon 23 skipping-treated *mdx* mice that had similar dystrophin levels as assessed by Western blotting [67]. Serum myomiR levels were restored to wild-type levels in the exon skipping-treated mice but surprisingly not in *mdx*-*Xist*^Δhs^ mice, highlighting differences between the two models.

A second point is that the expression pattern of dystrophin in the heart, and not just its level, appears to have a considerable impact on cardiac phenotype as well. Uniformly low dystrophin levels at 3.3% generally did not improve the cardiac phenotype in *mdx*^3cv^ mice, despite being near the 4% dystrophin level that was observed to ameliorate cardiac symptoms in mosaic models [75]. We have to recognize that although most cell and gene therapies for DMD have the same goal of increasing cardiac dystrophin levels, they may accomplish this by inducing different patterns of dystrophin expression in the heart. Cell therapy, micro/mini-dystrophin delivery, genome editing, and antisense therapy (e.g., exon skipping) usually lead to mosaic dystrophin expression. However, it may be possible to observe uniform dystrophin rescue in some instances [68]. A careful assessment of the dystrophin rescue pattern induced by such treatments is therefore recommended during pre-clinical study. Let us now consider genome editing and exon skipping in depth.

CRISPR-mediated genome editing to correct dystrophin mutations has emerged as a promising therapeutic approach for DMD [53,54]. The CRISPR system essentially consists of a CRISPR-associated (Cas) endonuclease that is guided by a short, single-stranded RNA molecule (called the guide RNA) to a site in the genome where it induces a double-stranded DNA break [54,89,90]. The approach can be tailored to remove single or multiple out-of-frame exons in the *DMD* gene. In a previous article, we have comprehensively reviewed pre-clinical studies using CRISPR to restore dystrophin protein levels in the heart [53]. One study showed that one-time treatment of 6-week-old female *mdx* mice with CRISPR/Cas9 to delete *Dmd* exon 23 led to 9% dystrophin of wild-type levels in the heart at 18 months by Western blotting, which resulted in improved ECG, EF, and hemodynamic function [91]. Another study demonstrated improved SV and cardiac output in male *mdx* mice 19 months after being treated with CRISPR/Cas9 to delete *Dmd* exons 21–23 at post-natal day 3 [92]. These mice had 2.16% dystrophin of wild-type levels in the heart by Western blotting, or 11.1% mosaic dystrophin-expressing CMs by immunostaining. Both these studies agree with findings from *mdx*-*Xist*^Δhs^ mice on the effectiveness of low levels of mosaic dystrophin expression in ameliorating dystrophic cardiac phenotypes.

Despite their success in pre-clinical studies, exon skipping with phosphorodiamidate morpholino oligomers (PMOs) to correct mutant *DMD* transcripts exhibit poor efficacy in the heart [88]. Thus, cell-penetrating peptides have been conjugated to PMOs (resulting in what we call PPMOs) in order to improve their uptake and efficacy in CMs [88,93]. Two studies reported ≈ 30% of wild-type dystrophin levels after exon 23-skipping PPMO treatment in *mdx* mice, which reduced inflammation and fibrosis in the heart, as well as prevented exercise-induced cardiac dysfunction [94,95]. Interestingly, one study showed that treatment with exon 23-skipping PPMOs could reduce the severity of cardiac hypertrophy in *mdx* mice 7 months after their last injection [96]. Since PPMO-induced dystrophin rescue in this study only lasted 11 weeks post-treatment, this meant that some amelioration of cardiac phenotypes was possible even though the hearts of treated mice lacked dystrophin for 4 months post-treatment. PPMOs have also been tested on the canine X-linked muscular dystrophy in Japan (CXMDJ) DMD model [97]. Intravenous administration of a PPMO cocktail skipping dystrophin exons 6–8 in two dogs increased dystrophin levels in the heart to 5% of wild-type by Western blotting. Vacuole degeneration in cardiac Purkinje fibers was reduced in these dogs compared to their non-treated counterparts, to levels seen in wild-type dogs. ECG abnormalities were ameliorated post-treatment as well. Upon examination of the myocardium, PPMO treatment in these dogs led to mosaic dystrophin expression. Overall, findings from these studies suggest that it is possible to observe improvements in dystrophic cardiac phenotypes with PPMO treatment, even with low levels of mosaic dystrophin rescue induced.

Going back to our earlier discussion, the third point we have to recognize from the mosaic carrier animal studies is that when these models do express dystrophin, it is of the wild-type, full-length form. Most gene therapies for DMD focus on restoring amounts of truncated dystrophin variants in vivo. Of relevance to the genome editing and exon skipping approaches, which are based mainly on the reading frame rule, observations from patient mutation databases indicate that not all in-frame deletions lead to the milder BMD phenotype [98,99]. In fact, an in-frame deletion may be associated with either DMD or BMD to varying degrees depending on the mutation. Thus, whether the shorter dystrophin variants used in gene therapies function similarly to the full-length protein remains an area of active research [100].

Finally, the last point to consider is that there are simply a myriad of factors that could have influenced the cardiac phenotype observed in animal models. Besides obvious differences in cardiac anatomy and physiology, there are fundamental genetic and environmental differences that exist between mice and humans. We have previously mentioned the study on hiPSC-derived EHMs, and that the presence of 30% dystrophin-rescued CMs could not fully improve cardiac contractility to healthy levels [63]. Although contractility measurements were not performed on isolated CMs of *mdx*-*Xist*^Δhs^ mice, it is in striking contrast to the improved cardiac function seen with at least 4% mosaic dystrophin expression. Even within mouse models there is a lot of disparity that has to be explained, e.g., with the differences in cardiac phenotype exhibited by mice from mixed *mdx*/wild-type blastocysts [71], or in mice of varying genetic backgrounds [68,70,74]. In summary, while findings from animal models are definitely helpful in determining the level of dystrophin rescue we should aim for in the heart, these results have to be interpreted with careful deliberation prior to extending their implications to the human case.

## 6. Conclusions

Understanding the causes and consequences of dystrophin deficiency in females is a steadily growing area of research. Cardiac involvement is an especially major concern, as it may progress into severe heart failure or at the very least pose certain health risks [18]. Although we are now getting a better idea of the genetic events leading to DMD/BMD symptoms in female carriers, we still could not explain or account for the wide phenotypic variability observed in these individuals [18,22,24]. More clinical studies are needed to establish genotype–phenotype correlations, which will also help fill in the information gap with regard to the prognosis, management, and treatment of manifesting female carriers. It would likewise be beneficial to more clearly elucidate the roles of dystrophin in the heart. Besides stabilizing CM cell membranes, dystrophin regulates the activity of other CM membrane proteins, particularly calcium ion channels [101,102]. As calcium has a central role in excitation–contraction coupling [103], this implies that dystrophin has a direct influence on heart pump function. In addition to full-length dystrophin, the shorter Dp71 dystrophin isoform is also expressed in the heart, specifically at the CM T-tubules [104]; how it influences cardiac function is unknown. Further research into these areas will provide insight into what extent dystrophin-positive CMs can compensate for their dystrophin-negative counterparts in the hearts of female carriers, and also facilitate the development of more effective management and therapeutic approaches for the disease.

In line with this, efforts to increase dystrophin levels in the heart are actively ongoing. Findings in animal models suggest that low levels of dystrophin are sufficient for maintaining at least some semblance of proper cardiac function [69,70]. This is supported by a number of reports that have tested genome editing and exon skipping therapies in vivo [91,92,96,97]. However, as we have seen, there are disagreements in the literature as to what amount of dystrophin expression is actually beneficial [67,71,105]. The issue is further complicated by the fact that therapies restore cardiac dystrophin levels in a diverse manner, e.g., with different truncated dystrophin variants produced, or different patterns of dystrophin expression induced. A systematic analysis of results from existing preclinical studies may help resolve this confusion. Nevertheless, there is a trend of better cardiac function with higher dystrophin levels in humans and animal models [69,70,105]. Continued work to enhance the efficacy of dystrophin-increasing therapies should ensure the successful treatment of dystrophic phenotypes in the heart and other affected tissues such as skeletal muscle. Ultimately, these studies will improve disease outcomes in patients with DMD/BMD, for both males and manifesting females.

## Figures and Tables

**Table 1:** Mouse models of female dystrophin mutation carriers.

Model	Type	Dystrophin % in Heart	Cardiac Phenotype	Ref/s
*mdx*/X	Mosaic	≈50%	Normal, similar to wild-type at 3 or 21 mos	[30,65]
*mdx*-*Xist*^Δhs^	Mosaic	Varies	Correlates with dystrophin level; mice with >4% dystrophin have normal heart function; fibrosis increased at 6, 10 mos regardless of dystrophin amount	[68,69]
*mdx*/*utrn*^−/−^/*Xist*^Δhs^	Mosaic	Varies	Correlates with dystrophin level; generally worse than *mdx*-*Xist*^Δhs^ mice at similar ages, as well as a reduced lifespan for mice with lower dystrophin levels	[70]
*mdx*/WT chimeras	Mosaic	Varies	Unexpectedly severe cardiac function with chimeras containing 30% or 50% *mdx* cells; mice with <5% *mdx* incorporation mostly wild-type	[71]
*mdx/mdx*	Uniform	0%	BL10 background: more severe hemodynamic function and EF at 22 mos than male *mdx* mice, as well as having increased heart mass; D2 background: lower heart mass than male *mdx* mice at 8 mos	[72,73,74]
*mdx* ^3cv^	Uniform	≈3.3%	Anatomical and histopathological features no different from dystrophin-null *mdx*^4cv^ mice; diastolic function improved, but not overall cardiac function compared to *mdx*^4cv^ mice	[75]
